# Effect of a Mobile App-Based Exercise Program on Diastasis Recti Abdominis, Muscle Strength, Anthropometric Measures, and Satisfaction Among Post-Cesarean Primiparous Mothers: A Randomized Controlled Trial

**DOI:** 10.3390/healthcare13233103

**Published:** 2025-11-28

**Authors:** Gehan A. Abdelsamea, Shimaa Abdelalim Essa, Azza Sayed Abdelrehim Khalil, Hoda M. Zakaria, Rehab S. Mamoon, Mohamed G. Ali

**Affiliations:** 1 Department of Physical Therapy for Women’s Health, Faculty for Physical Therapy, Cairo University, Giza 12613, Egypt; 2 Department of Basic Sciences, Faculty for Physical Therapy, Suez University, Suez 43221, Egypt; s.essa@the.suezuni.edu.eg; 3 Department of Rehabilitation Sciences, College of Health and Rehabilitation Sciences, Princess Nourah bint Abdulrahman University, P.O. Box 84428, Riyadh 11671, Saudi Arabia; askhalil@pnu.edu.sa; 4 Department of Physical Therapy for Neurology, Faculty for Physical Therapy, Cairo University, Giza 12613, Egypt; dr.hodazakaria@cu.edu.eg; 5 Department of Physical Therapy for Women’s Health, Faculty for Physical Therapy, South Valley (Qena) University, Qena 33523, Egypt; dr_m.gamal1987@svu.edu.eg

**Keywords:** abdominal exercises, cesarean birth, diastasis rectus abdominis, inter-rectus separation, manual muscle testing, mobile application

## Abstract

**Highlights:**

**What are the main findings?**

- A mobile app-based abdominal exercise program (“Just Fit”) significantly reduced inter-recti distance (IRD) above the umbilicus and improved abdominal muscle strength more effectively than traditional exercise in post-cesarean primiparous mothers.
- Both mobile app-based and traditional exercise programs produced significant improvements in IRD, abdominal girth, and muscle strength after 8 weeks, confirming their value in managing diastasis rectus abdominis (DRA).

**What are the implications of the main findings?**

- The validated mobile application provides an effective, accessible, and engaging tool for physiotherapists to deliver postpartum rehabilitation and monitor outcomes remotely.
- Integrating digital health technologies into women’s post-cesarean recovery programs can enhance adherence, satisfaction, and clinical effectiveness in DRA management.

**Abstract:**

**Background**: Diastasis rectus abdominis (DRA) is a frequent concern following childbirth, particularly following a cesarean section (CS). Mobile exercise applications offer promising opportunities for enhancing physical therapy services, with potential positive outcomes. **Purpose**: This study compared the effect of a specific DRA-targeted mobile app-based exercise program on inter-recti distance (IRD) and multiple clinical measures to a traditional abdominal exercise program in post-CS mothers with DRA. **Methods**: This two-armed, parallel-group, randomized controlled trial involved 40 primiparous women undergoing CS; they were classified into two equal groups. Group A participated in Just Fit, a mobile app-based abdominal exercise program, while Group B received a traditional abdominal exercise program. Both exercise programs lasted 30 min, 3 times/week, for 8 weeks as a home program with follow-up sessions once weekly at an outpatient physical therapy clinic. Ultrasonography was used to measure IRD, a manual muscle test assessed abdominal muscle strength, a tape measure gauged circumferences, and a questionnaire evaluated satisfaction. **Results**: Both programs showed significant improvements in pre- and post-treatment measures of IRD, muscle strength, and girth (*p* ≤ 0.001 for all comparisons). Women in Group A exhibited significant post-treatment improvements in IRD above the umbilicus, abdominal muscle strength, girth measurements, and satisfaction compared with those in Group B. However, there were non-significant differences in IRD below the umbilicus and hip circumference between the two groups. **Conclusions**: The mobile app-based exercise program was associated with greater improvements in IRD above the umbilicus, abdominal muscle strength, waist and umbilical circumferences, and patient satisfaction compared with the traditional exercise program for post-CS DRA. These findings suggest that mobile app-guided rehabilitation may serve as an effective and accessible adjunct to traditional post-CS exercise programs, although larger trials are recommended to confirm these results.

## 1. Introduction

Diastasis of the recti abdominis (DRA), also known as rectus diastasis, refers to the separation of the two sections of the rectus abdominis muscle along the midline of the linea alba without any macroscopic defect in the fascia [1]. DRA is most common during and after pregnancy. The documented prevalence of DRA differs across studies and can be inaccurate as a result of differing intra-rectus distance (IRD) cutoff values for establishing its diagnosis. This variation is also influenced by different assessment methods (such as palpation, calipers, computed tomography, or ultrasonography) and whether measurements are taken at rest or during activity. Moreover, the measurement sites (whether single or multiple locations above, below, or even at the umbilicus), and some personal factors like a history of diminished abdominal muscle strength, high body mass index (BMI), pelvic floor dysfunctions, and pelvic girdle pain can contribute to inconsistencies [2,3,4]. The prevalence of DRA is 66–100% throughout the third trimester, 60% at the 6th week postpartum, and 32.5% at the 12th month postpartum [5,6].

Cesarean section (CS) is a delivery mode that involves the expulsion of one or more fetuses through both abdominal and uterine incisions [7]. The lower transverse abdominal approach is likely the most common method used for cesarean delivery [7], which involves making an incision through the myofascia of the external and internal abdominal obliques and transversus abdominis, thereby potentially contributing to rectus abdominis separation [8]. Nowadays, CS is among the most commonly conducted surgical operations on women. Data from 150 countries indicate that approximately 18.6% (ranging from 6% to 27.2%) of all births are delivered via CS, with an average annual growth rate of 4.4% [9]. Factors contributing to this rise include advanced maternal age, improved economic and social conditions, and misconceptions regarding the safety of vaginal delivery [10]. Although CS has been strongly linked to postpartum musculoskeletal conditions such as low back pain [11,12,13,14,15,16], it is more specifically associated with significant postoperative alterations in the abdominal fascia and muscle thickness. In contrast, vaginal delivery predominantly affects muscle thickness alone. Ultrasound assessments following CS reveal features such as rectus abdominis thinning, an increased IRD, and thickening of the linea alba, all of which may contribute to the development of DRA. Consequently, the mode of delivery appears to influence the incidence of postpartum DRA, with a higher prevalence observed in women who underwent CS compared to those who had vaginal delivery [17,18]. Due to this increased risk, the present study focused exclusively on mothers who delivered via CS.

Management of DRA includes, firstly, its diagnosis and then its targeted interventions. Diagnosing DRA typically involves manual palpation and estimating the gap or distance between the two recti abdominis along the linea alba’s tendinous sheet. Modern imaging methods like magnetic resonance and ultrasonography are now more commonly used to precisely measure the extent and dimensions of this separation, offering crucial details for planning interventions [19]. Interventions for DRA may be through conservative or surgical approaches. The conservative approach focuses on physical therapy and personalized exercise regimens. This approach aims to strengthen specific deep local core muscles like the transverse abdominis along with the pelvic floor, demonstrating positive outcomes in decreasing the gap between the rectus abdominis muscles and enhancing overall functional recovery [20].

Mobile app-based exercises offer convenient access to customized fitness routines that suit individual preferences and schedules. These apps provide flexibility in workout timing and location, accommodating individuals, especially women, who prefer exercising at home and those who can benefit by integrating workouts seamlessly into their daily schedules without the need for travel to a gym or clinic [21]. Additionally, mobile app-based exercise programs can effectively ensure consistent engagement in fitness activities [22]. Research indicates that these programs yield substantial health benefits comparable to traditional supervised settings. They offer personalized workout plans, real-time feedback, and motivational cues that enhance adherence and improve outcomes [23].

Although mobile exercise programs are increasingly used in general fitness and rehabilitation, there is limited empirical evidence evaluating their effectiveness on post-CS DRA recovery. This gap is likely due to the specialized needs and safety considerations of post-CS women, the absence of standardized postpartum exercise protocols, and the wide variability in DRA severity and healing patterns. Additionally, challenges in recruiting and retaining postpartum participants, the need for in-person assessments to accurately measure DRA, and the lack of validated, clinically designed mobile exercise content further constrain research in this area. As mobile health technologies for postpartum rehabilitation are still emerging, rigorous clinical trials have not yet kept pace with their rapid development and widespread use. So, this study aims to be the first RCT to explore the efficacy and convenience of app-based exercises in promoting the recovery of DRA in post-CS mothers, improving abdominal muscle function, and enhancing patient satisfaction. Its findings have the potential to influence clinical practice by offering a convenient and accessible alternative to traditional rehabilitation methods.

The Null Hypothesis stated that there would be a non-significant effect of mobile-app-based exercises on DRA, and that there would be non-significant improvements in secondary outcomes such as abdominal muscle strength, anthropometric measures, and satisfaction in post-CS mothers.

## 2. Materials and Methods

### 2.1. Trial Design

The study design is a two-armed, parallel-group, randomized controlled trial conducted from April 2023 to October 2023. The study examines the effectiveness of a mobile app-based abdominal exercise program (JustFit) compared with a traditional abdominal exercise program, both targeting DRA rehabilitation. Both were home-based programs with identical session frequency and duration, differing only in their delivery mode and content structure (mobile-guided vs. therapist-prescribed traditional routine).

### 2.2. Study Participants and Sample Size

A very large effect size for abdominal exercises on IRD was reported by Awad et al. [24], who found an effect size of 2.6 for a program combining progressive abdominal plank training, DRA-related advice, and abdominal binder use compared with a control group that received only advice and a binder. Their use of ultrasound to measure IRD aligns with the primary outcome measure in the present study. Similarly, Mahalakshmi et al. [25] reported a very large effect size of 1.5 for targeted abdominal exercise interventions. Based on these findings, an alpha level of 0.05, a power of 80%, and the rationale for selecting a large effect size consistent with previous rehabilitation trials demonstrating strong localized effects of exercise on DRA, a total of 40 primiparous post-CS women were sufficient for this study. They had diastasis recti measuring ≥two finger widths upon palpation and exhibited a bulging abdomen. These women were referred by an obstetrician to the outpatient physical therapy clinic at Delta University for Science and Technology, Egypt, where the study was conducted. The primary outcome was IRD, whereas the other measured variables were considered secondary outcomes.

Inclusion criteria were specific to first-time mothers who delivered exclusively by cesarean section, aged from 25 to 35 years, and whose body mass index (BMI) ranged from 24.5 to 29.4 kg/m^2^. They also had diastasis recti of at least 1 cm above the umbilicus and at least 0.5 cm below it. Exclusion criteria comprised mothers who had vaginal deliveries, multiparous mothers, those without diastasis recti, mothers younger than 25 or older than 35, and those with a BMI less than 24.5 or greater than 29.4.

**Figure 1:**
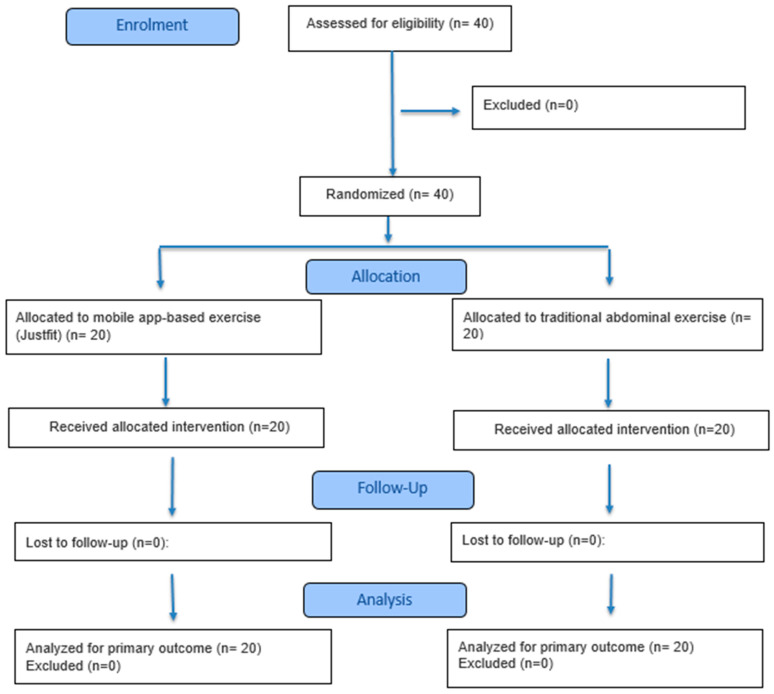
CONSORT 2025 flow diagram.

This randomized controlled trial is reported according to the CONSORT 2025 guidelines, ensuring transparent and comprehensive communication of all major trial design and results elements. The study was registered at ClinicalTrials.gov (NCT07001046) and follows ethical and reporting standards for randomized trials. The design, conduct, analysis, and reporting of the trial have been prepared in accordance with the CONSORT 2025 Statement [26], including the standard 30-item checklist and participant flow diagram (Figure 1).

The program began 12 weeks after each participant’s delivery and lasted for 8 weeks. The 40 women participating in the study were randomly classified into 2 groups, each containing an equal number of participants. Group A: 20 primiparous women participated in JustFit (version 1.6.6) a mobile app-directed exercise program for the abdominal muscles, which was downloaded free of charge from the Google Play Store during their initial visit to complement their treatment. Group B: 20 primiparous women received a traditional abdominal exercise program.

### 2.3. Randomization Procedure

Once eligibility was verified, the 40 participants were randomly assigned to two equal groups (20 women each) using a simple randomization technique. A computer-generated table of random numbers was used to allocate participants to either the experimental (study) group or the control group. The randomization sequence was prepared by an independent researcher who was not involved in participant assessment or treatment administration. To ensure allocation concealment, group assignments were placed in sequentially numbered, sealed, opaque envelopes. At the time of enrollment, each participant selected one envelope to determine her group assignment. Blinding was not feasible due to the nature of the intervention (mobile vs. traditional exercise). However, outcome assessments like ultrasonographic IRD measurement were conducted by a radiologist blinded to group assignment to minimize assessment bias.

### 2.4. Examination and Instrumentation

There is no consensus on the size of IRD classified as pathological RDA. Various measurements are provided: greater than 1 cm above the umbilical level, over 2.7 cm at the umbilical level, and greater than 0.9 cm below the umbilical level for individuals under 45 years old; in individuals over the age of 45, the respective values are 1.5 cm, 2.7 cm, and 1.4 cm [27]. Another study suggests diagnosing diastasis when the IRD is more than 1.4 cm above the umbilical level, more than 2 cm at the umbilical level, and over 0.2 cm below the umbilical level [28]. This study used the measurement guidelines suggested by Ruth et al. [25] to identify the presence of DRA due to their age-specific criteria.

#### 2.4.1. Ultrasonography

Ultrasonography is a valid tool for assessing DRA, showing a moderate correlation (r = 0.71) with calipers [29]. It also demonstrates good reliability, with an inter-rater ICC of 0.86 and an excellent intra-rater ICC of 0.96 [30].

To measure inter-recti distance, the radiologist used the Philips HD15 Ultrasound healthcare system (Amsterdam, The Netherlands) using an L12-4 broadband linear array transducer at a frequency of 16 Hz at a depth of 4–6 cm. The participants were placed in a comfortable supine position to measure the IRD. Ultrasound coupling gel was applied to the imaging sites to provide good acoustic interaction between the transducer and the overlying skin. The transducer was initially positioned perpendicular to the linea alba, above the umbilical level at the midpoint between the umbilicus and the xiphoid process. It was subsequently relocated to the umbilical level and then placed below the umbilicus, halfway between the umbilicus and the symphysis pubis. The focal scope was adjusted to encompass the medial aspects of both recti. During normal expiration, the hyperechoic linea alba and the hypoechoic bilateral rectus abdominis muscles were detected to measure the IRD.

#### 2.4.2. Manual Muscle Testing

Manual muscle testing (MMT) is used to reliably assess muscle strength by medical, chiropractic, osteopathic, athletic training, physical therapy, and rehabilitation professionals [31,32]. A half curl-up is conducted to evaluate abdominal muscle strength, either the rectus abdominis or the abdominal obliques. To assess the rectus abdominis, the exercise starts with the patient lying on their back, feet unsupported. The patient tilts their pelvis backward to flex the lumbar spine, then flexes the cervical spine, followed by the thoracic spine, lifting the head and scapulae off the surface. Performing the curl-up movement with the feet unsupported is more effective at activating the rectus abdominis muscle than executing a full sit-up from a supine position with the feet supported [33]. Also, only 45° trunk flexion is required to assess pure trunk flexion for the rectus abdominis muscle. To assess the abdominal oblique muscles, the researchers apply the same procedure but only with the patient’s movement toward trunk flexion with rotation.

The utilized grading system according to Clarkson 2013 [34] is presented in Table 1:

#### 2.4.3. Girth Measurements

Girth measurements measured at both the waist and umbilical levels are valid indicators of visceral fat for both women and men [35]. Moreover, girth measurements for the waist and hip are highly reliable methods for assessing the body’s circumferences, with ICCs of 0.97 and 0.96, respectively [36].

Girth measurements refer to the circumferential assessments taken at specific anatomical locations on the body. These measurements are obtained using a tape and are useful for assessing body size and tracking changes in these aspects [37]. The location to be measured should be marked. When measuring, ensure the tape is neither too loose nor too tight. It should lie flat against the skin and be positioned horizontally. The individual should stand comfortably upright with their hands at their sides, without contracting the abdominal muscles.

Based on guidelines of the American College of Sports Medicine in 2023, the researcher measured the waist circumference at the narrowest part of the torso above the umbilicus, usually between the bottom of the ribs and the iliac crest. The abdominal (umbilical) girth was measured by taking the perimeter distance around the torso at the level of the umbilicus. For the hip circumference, the researcher measured the widest part of the buttocks above the gluteal folds [38].

#### 2.4.4. Measure Yourself Medical Outcome Profile (MYMOP)

The MYMOP is a concise, patient-derived, problem-focused survey that asks individuals to identify the symptom that bothers them the most (in this study, it was DRA). Then, participants assess their level of satisfaction with the treatment method, like the mobile-app-based exercise program in this study.

**Figure 2:**
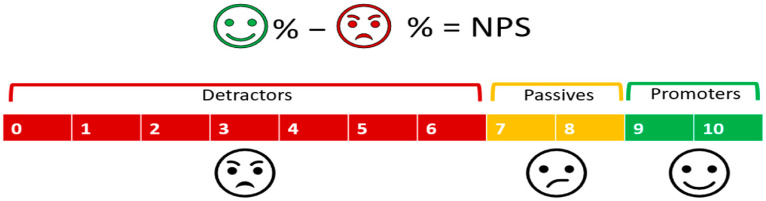
A clarification of how the NPS is calculated [39].

To assess the satisfaction level, the Net Promoter Score (NPS) was calculated by asking women to answer the following final question, “Would you consider recommending this program to a colleague or friend?”, on the scale provided in Figure 2.

Due to the lack of variance in this variable, to analyze the factors associated with Promoter status, we generated a binary variable assigned a value of 1 if the participant scored 9 or 10 on the NPS item, and zero otherwise [40].

### 2.5. Therapeutic Interventions

**Figure 3:**
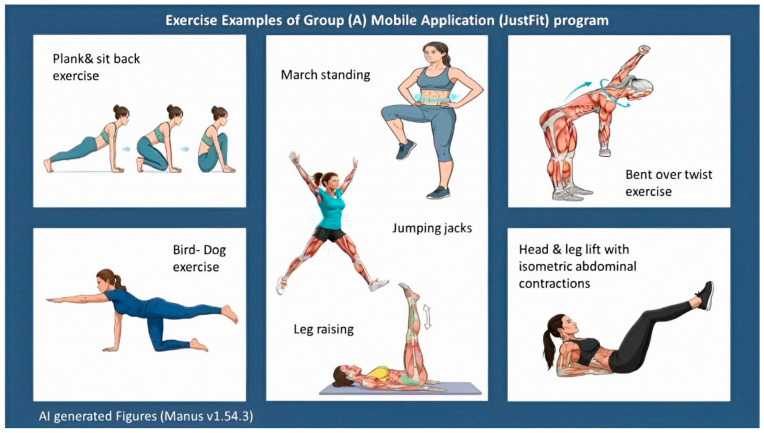

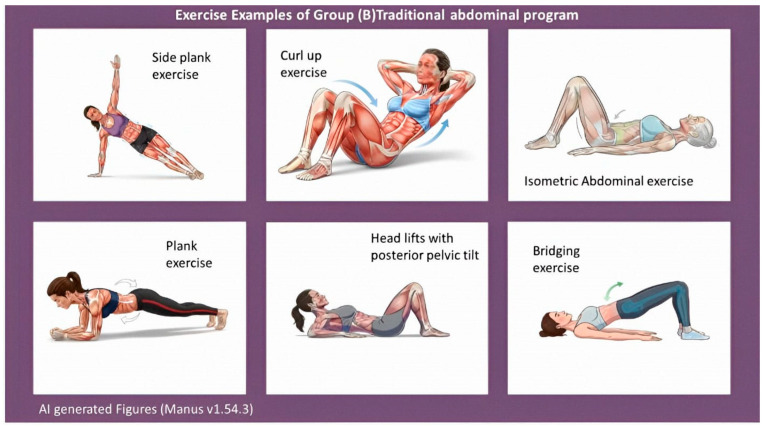
Examples of both exercise programs.

Participants in Group A performed an individualized exercise program using the JustFit mobile application (ENERJOY PTE. Ltd., Singapore) (Figure 3). While, participants in Group B followed a standardized abdominal exercise protocol (Figure 3). The description of both exercise programs is illustrated in Table 2.

### 2.6. Ethics Statement

This study was authorized by the Research Ethical Committee of the Faculty of Physical Therapy at South Valley (Qena) University under No: P.T -GYN-07/2025-571 on 29 July 2025. This study has a registration number of ID: NCT07001046 on ClinicalTrials.gov (retrospective registration on 3 June 2025). An informed consent form was taken before enrollment.

### 2.7. Data Collection and Statistical Analysis

The Shapiro–Wilk test was employed to assess the normality assumption of the data. The results of the Shapiro–Wilk test indicated that certain variables, such as the body mass index (BMI) and age, demonstrated a normal distribution (i.e., *p* > 0.05). As a result, a parametric analysis was conducted for these variables. Conversely, other variables, including pre- and post-treatment IRD above the umbilicus, pre- and post-treatment IRD below the umbilicus, pre- and post-treatment MMT for rectus abdominis, pre- and post-treatment MMT for abdominal obliques, pre- and post-treatment waist circumference, pre- and post-treatment umbilical circumference, pre- and post-treatment hip circumference, and post-treatment satisfaction level, exhibited a non-normal distribution (i.e., *p* ≤ 0.05). Consequently, a nonparametric analysis was conducted for these variables. Regarding the statistical analysis, we utilized the IBM SPSS Statistics for Windows version 25 to execute the following statistical procedures:

Descriptive Statistics for the normally distributed variables; the statistical measures used were the mean and standard deviation (SD). On the other hand, for the non-normally distributed variables, the statistical measures used were the median and interquartile range [IQR].

The independent *t*-test was employed for mean comparisons between the two groups for variables that follow a normal distribution.

The Wilcoxon Test was utilized for pairwise comparison of the mean ranks in the same group for different times of measurement (pre- and post-treatment) for the non-normally distributed variables.

The Mann-Whitney test was performed for mean rank comparisons between the two groups for variables that did not follow a normal distribution.

The rank-biserial correlation (rb) was calculated as an effect size measure. This coefficient was selected because it is specifically appropriate for data analyzed with the nonparametric tests, does not require distributional assumptions, and provides an interpretable index of the magnitude and direction of group differences ranging from −1 to +1.

The post hoc power analysis was performed using a Monte Carlo simulation-based approach to determine whether the study had adequate power to detect the observed effect sizes. This analysis was conducted to assess the reliability of statistically significant findings, evaluate the risk of Type II error, and confirm that the achieved sample size was sufficient for the nonparametric outcomes examined.

Spearman’s Correlation test was performed to measure the relationship between post-treatment IRD above and below the umbilicus and the other post-treatment variables.

The Multi-linear Regression Analysis was performed to examine the independent effects of IRD above and below the umbilicus on post-treatment abdominal muscle strength and circumference measures, while adjusting for potential confounding variables such as age and BMI.

Multiple pairwise tests were performed without formal correction.

Statistical significance was set at the 0.05 level (*p* ≤ 0.05) for all analyses.

## 3. Results

### 3.1. Physical Features of Participants

The mean ± SD BMI values for Groups A and B were 27.31 ± 1.58 and 27.41 ± 1.19 kg/m^2^, respectively, with a negligible effect size (d = −0.072). The mean ± SD ages were 28.45 ± 1.79 and 28.55 ± 2.78 years, also showing a negligible effect size (d = −0.042). Independent *t*-tests revealed no significant differences between the two groups in BMI or age (*p* = 0.823 and 0.893) (Table 3).

### 3.2. The Wilcoxon Test for Pairwise Comparison of the Same Group (Pre- and Post-Treatment), and the Mann–Whitney Test for Comparisons Between the Two Groups

#### 3.2.1. Regarding the Ultrasonographic Findings of IRD

For IRD above the umbilicus, both groups had identical pre-treatment medians (1.32 cm) with negligible between-group effect size (rb = 0.03). Post-treatment, Group A improved to 0.89 cm compared with 1.10 cm in Group B, and the between-group difference became significant (*p* < 0.001) with a very large effect favoring Group A (rb = −0.972). Within-group changes were significant and showed very large effect sizes in both groups. Post hoc power was 100%.

For IRD below the umbilicus, pre-treatment medians were 1.58 cm (Group A) and 1.44 cm (Group B), with a small-to-moderate effect size (rb = 0.248) and no significant difference (*p* = 0.178). Post-treatment values were 1.19 cm and 1.30 cm, respectively, again without a significant between-group difference (*p* = 0.122), although the effect size remained small to moderate (rb = −0.285). Both groups showed significant pre- to post-treatment improvements with very large within-group effect sizes. Post hoc power was 85% (Table 4).

#### 3.2.2. Regarding Manual Muscle Testing (MMT) Variables

Both groups showed identical pre-treatment MMT scores for the rectus abdominis and abdominal obliques (median 4 [IQR 0]), with no significant between-group differences (*p* = 1.000). Post treatment, Group A improved to a median of 8 while Group B reached 7 for both muscle groups, resulting in significant between-group differences (*p* < 0.001). Within-group improvements were also significant (*p* < 0.001). Rank-biserial correlations demonstrated a perfect effect size favoring the mobile-app-based exercise program (rb = −1.000; 95% CI: −1.000 to −0.980), and post hoc power analysis confirmed 100% power (Table 5).

#### 3.2.3. Regarding Girth Measurements at Different Levels

Pre-treatment waist, umbilical, and hip circumferences were comparable between groups (*p* > 0.05). Post-treatment, both groups showed significant within-group reductions in all circumferences (*p* < 0.001). Between groups, the mobile-app group demonstrated significantly greater reductions in waist (*p* = 0.006) and umbilical circumference (*p* = 0.010), supported by moderate effect sizes (waist: rb = 0.502; umbilicus: rb = 0.470). Hip circumference did not differ significantly between groups post-treatment (*p* = 0.335), with a negligible effect size (rb = 0.041). Post hoc power analyses indicated excellent power for all measures (89–94%) (Table 6).

#### 3.2.4. Regarding the Post-Treatment Satisfaction Level

Post-treatment satisfaction scores were higher in Group A (median 1 [IQR 0]) than Group B (median 0 [IQR 1]), with a significant between-group difference (*p* = 0.003). The mobile-app group showed a moderate effect size (rb = −0.450; 95% CI: −0.700 to −0.090). Post hoc power was 70% (Table 7).

#### 3.2.5. Regarding Measuring Correlations

Spearman correlation analysis indicated that IRD above the umbilicus post-treatment was strongly negatively correlated with muscle strength measures, including MMT rectus abdominis (ρ = −0.84, *p* = 0.000) and MMT abdominal obliques (ρ = −0.84, *p* = 0.000), suggesting that lower IRD is associated with greater muscle strength. IRD above the umbilicus also showed moderate positive correlations with waist circumference (ρ = 0.43, *p* = 0.006) and umbilical circumference (ρ = 0.32, *p* = 0.047). In contrast, IRD below the umbilicus had weaker non-significant correlations with these variables (Table 8).

#### 3.2.6. Regarding Measuring Regressions

The multiple linear regression analyses adjusting for age and BMI confirmed that IRD above the umbilicus remained a significant negative predictor for both muscle strength outcomes (β = −2.76 for MMT rectus abdominis; β = −2.76 for MMT abdominal obliques, both *p* = 0.000), independent of covariates. IRD above the umbilicus also positively predicted waist and umbilical circumferences, though the explained variance was modest (R^2^ approximately 0.1 to 0.13). Coefficients for IRD below the umbilicus were generally smaller and non-significant after adjustment. Age and BMI also showed independent effects on some outcomes, emphasizing the importance of adjusting for these factors (Table 8).

## 4. Discussion

Physiotherapy is the primary treatment for DRA. Surgery should be considered only for patients with functional impairment and only after they have completed standardized six-month deep abdominal core exercises [41]. Various health-related mobile apps demonstrated good psychometric properties when used as substitutes for certain paper-based data-collecting instruments and to measure specific physical activity metrics. These applications can offer positive gains when used to provide physical training or exercise interventions [42]. This study aimed to compare various clinical parameters between mothers in the two groups and assess the impact of a specific DRA-targeted mobile app-based exercise program on these parameters. The results provide valuable insights into each group’s exercise program’s effectiveness.

The baseline features of the participants, including BMI and age, were similar between the two groups, besides all pre-treatment measures, suggesting that any observed differences in the outcomes can be attributed to the exercise program rather than differences in baseline characteristics.

Ultrasonographic findings of inter-recti separation showed significant improvements in the IRD both above and below the umbilicus for both groups (comparing pre- and post-treatment within each group). Post-treatment measures between the two groups indicated a significant difference above the umbilicus in mothers of the mobile application group compared to those of the traditional exercise program. Having 100% statistical power for the IRD above the umbilicus between groups means the study is virtually certain to detect any true effect of the intervention on this outcome if it exists. This eliminates the risk of a Type II error (false negative), ensuring that the observed significant difference post-treatment reflects a genuine effect rather than being a chance finding or due to inadequate sample size. However, there was a non-significant difference below the umbilicus. This suggests that the mobile app-based exercise program applied to Group A was more effective in reducing IRD above the umbilicus rather than below it. The post hoc power estimation of 85% for IRD below the umbilicus is highly sensitive to detecting true effects if they exist, meaning a non-significant result is less likely due to insufficient sample size or chance, and enhancing confidence that the null finding is valid.

In this study, the improvement of IRD medians above the umbilicus, both at the baseline and after both interventions, was greater than the improvement below the umbilicus. Some contributing factors may support the earlier recovery of DRA above the umbilicus compared to below it: (1) With the progression of pregnancy, uterine growth first elongates the muscles of the lower abdominal wall, often leading to greater separation below the umbilicus [43]. (2) Anatomically, the area above the umbilicus typically has more rectus abdominis muscle mass and fewer connective tissues compared to the area below the umbilicus, which makes the upper section of the rectus abdominis more responsive to strengthening exercises [44]. (3) Microscopically, the linea alba tends to be thinner and less resilient below the umbilicus, and this difference in connective tissue strength can make it more difficult to achieve significant improvements in diastasis recti in the lower abdomen [45]. Although these morphological differences between above and below the umbilicus are similar in both groups, the fair focus of the mobile application on exercise types targeting the upper abdominal muscles may be an acceptable interpretation of the exclusive significant improvement above the umbilicus.

This study reported decreases in IRD in both groups from pre- to post-treatment. These findings are consistent with prior research. For example, Kazmi et al., 2021 [46], demonstrated that abdominal exercises significantly reduced IRD both above and below the umbilicus in postpartum mothers. Similarly, Kim et al., 2022 [47] found that exercise interventions delivered through modern platforms such as real-time videoconferencing effectively improved IRD, quality of life, and trunk stability in postpartum women, highlighting their potential as alternatives to in-person sessions. Furthermore, Saleem et al., 2021 [48] reported a significant reduction in inter-recti distance among postpartum women who performed either upper or lower abdominal exercises, supporting the efficacy of both targeted and general core strengthening approaches. On the other hand, the research of Mota et al. 2015 [49] disagrees with our findings; they found that standard exercise programs like drawing-in exercises had minimal impact on inter-recti distance, particularly above the umbilicus.

The results of this study demonstrated significant improvements in abdominal muscle strength, as indicated by increased MMT scores for both the rectus abdominis and abdominal obliques in both groups, suggesting that both the mobile app-based and traditional exercise programs were effective. However, the post-treatment scores were significantly higher among mobile app users, indicating a superior effect of the app-based intervention in enhancing muscle strength. This superior effect may be attributed to several factors: the mobile app provided structured, progressive, and easily accessible exercise routines, incorporated reminders and guidance to ensure proper technique, and allowed participants to engage with exercises more consistently and independently, enhancing overall adherence. These findings are consistent with Keshwani et al., 2021 [50], who reported significant improvements in trunk flexor strength, measured by MMT, following postpartum exercise therapy, with or without abdominal binding. Collectively, these results emphasize the importance of well-structured, core-focused exercise programs in restoring abdominal muscle function after delivery and highlight how technology-supported interventions may further optimize outcomes.

Also, girth measurements at various levels (waist, umbilical, and hip circumferences) showed significant reductions post treatment for both groups (comparing pre- and post-treatment within each group). However, significant differences between the two groups post treatment were observed only for the waist and umbilical circumferences, not for hip circumference. These results suggest that the mobile application exercises were more effective in reducing central adiposity and weight loss compared to the traditional abdominal exercises. Our findings are supported by the findings of Turner-McGrievy et al., 2013 [51], who confirmed the potential benefits of mobile applications that offer physical activity programs besides dietary monitors in weight loss. Also, our findings align with the findings of Geusens et al. 2024 [52], which indicated that postpartum mothers who gain excessive weight during pregnancy may benefit from using smartphone app-based lifestyle interventions (physical activity and behavioral modifications), which help them reduce postpartum weight.

The median and interquartile range values for post-treatment satisfaction levels indicated significantly higher satisfaction in the group of the mobile app-based exercise program when compared to the group of traditional abdominal exercises. Our results align with those of Kwon et al., 2022 [53], who demonstrated higher satisfaction among users who utilized validated mobile applications for physical fitness following the COVID-19 crisis.

The results of the correlation analysis demonstrated a robust correlation between reduced IRD above the umbilicus and improved abdominal muscle strength following treatment, confirming the clinical relevance of targeted interventions to reduce IRD in this region. The persistence of this relationship after controlling for age and BMI supports its independent role beyond general physical features. The weaker correlations for IRD below the umbilicus suggest potential regional differences in the functional impact of IRD. Moderate positive correlations with waist and umbilical circumferences align with expectations that larger abdominal girth may accompany greater IRD, though the clinical implications require careful interpretation given modest effect sizes. The findings underscore the need for further longitudinal studies to clarify causal pathways and assess the utility of IRD as a therapeutic target.

The multiple linear regression analysis revealed that IRD above the umbilicus post-treatment is a significant independent predictor of abdominal muscle strength, with higher IRD associated with lower muscle strength even after adjusting for age and BMI. This highlights the clinical importance of targeting IRD reduction to improve functional outcomes. Waist circumference was also positively correlated with IRD and influenced by both age and BMI, reflecting the interplay between abdominal structure and body composition factors. The relatively low explained variance for hip circumference suggests additional unmeasured factors influence this measure, indicating a need for broader models in future research. These findings support the value of considering demographic and anthropometric covariates in abdominal health studies and suggest IRD above the umbilicus is a critical region for therapeutic focus.

This study has several strengths: First, this study is, to our knowledge, the first of its kind to examine the effect of an exercise-based mobile application on multiple integrated outcomes in post-CS mothers complaining of DRA. Second, it emphasizes valid and reliable measurement tools like ultrasonography to measure the primary outcome (IRD). Third, by comparing the effectiveness of mobile app-based exercise programs to traditional exercise programs, the study offers valuable insights into different rehabilitation approaches for DRA that help in identifying the most effective intervention. Fourth, by assessing multiple clinical parameters, including IRD, muscle strength, girth measurements, and satisfaction level, it provides a holistic view of the effectiveness of the interventions. Fifth, the statistical analysis employed rank-biserial correlation for effect size estimation, which is a robust nonparametric measure well-suited for the ordinal and non-normally distributed data typical in clinical and satisfaction assessments. Alongside, conducting a post hoc power analysis using a conservative Monte Carlo simulation method adds rigor by quantifying the study’s sensitivity to detect observed effects, improving transparency in the interpretation of findings. Sixth, this study measured the strength of the relationships between variables by Spearman’s correlation coefficients, while the independent predictive effects were quantified using multiple linear regression coefficients adjusted for relevant covariates to control for confounders.

This study also has several limitations: First, all participating postpartum women in this study underwent CS, which can restrict the generalizability of its findings because it may not apply to other women with DRA, such as pregnant women or those who experienced vaginal delivery. Second, the study lacks a follow-up period, which limits the understanding of the long-term sustainability of the improvements observed. Third, although MMT is a valid and widely used clinical tool, it remains partially subjective. The lack of instrument-based measures, such as dynamometry, limits the objectivity of muscle strength assessment. Future studies are encouraged to combine MMT with quantitative electromyography or dynamometric evaluations. Fourth, although multiple outcomes were analyzed, the study primarily focused on pre-specified variables (IRD, MMT, circumferences, and satisfaction). Therefore, the results should be interpreted with caution. Additionally, the modest sample size may limit the generalizability of the findings. Future studies with larger samples should incorporate Bonferroni or False Discovery Rate (FDR) corrections to reduce the risk of type I error (false positive results). Fifth, although body weight and height were measured during the pre-treatment assessment to calculate BMI, pre–postpartum weight changes were not recorded. As natural weight loss may occur after childbirth, this factor could have influenced the interpretation of changes in abdominal and waist circumferences, making it difficult to isolate the effects of the intervention. Sixth, despite all participants completing the 8-week study period and consistently reporting during follow-up visits that they adhered to their exercise programs, adherence was not formally measured or documented by the physical therapist; therefore, an objective adherence rate could not be determined.

The findings of this study provide insight into Women’s Health Physiotherapists’ perspectives on using modern technology, such as mobile app-based exercise programs, for post-cesarean care of DRA, with a focus on safe abdominal muscle strengthening exercises. In the context of global crises, such as COVID-19, the use of these validated mobile applications highlights their clinical significance by promoting patient education, saving time and costs, and allowing programs to be customized to individual patient needs and abilities. But, because the exercise program was generated by artificial intelligence, supervision by a healthcare provider remains essential to ensure correct performance and participant safety. Future studies with larger sample sizes are needed to provide a more comprehensive evaluation of muscle function by incorporating objective assessments alongside MMT, and long-term follow-up to examine the sustainability of outcomes. Future studies are recommended to include objective adherence tracking to both interventions for better interpretation of intervention effectiveness. Additionally, pre–postpartum weight assessments should be included in the forthcoming studies to control for this potential confounding variable.

## 5. Conclusions

The findings of this study demonstrate that both therapeutic approaches were effective in improving IRD above the umbilicus, muscle strength, and girth measurements. However, within the limits of this sample, adherence to the mobile-app-based exercise program produced remarkably superior and clinically meaningful improvements across all examined outcomes compared to the traditional abdominal exercise program, except hip circumference and IRD below the umbilicus, where the latter showed clinically meaningful but not statistically significant improvement. Furthermore, mothers who followed the mobile-app-based program reported higher levels of satisfaction. Focused supervision by a healthcare provider is critical for all mobile application-based exercise programs to ensure correct performance and participant safety.

## Figures and Tables

**Table 1:** MMT grading of abdominal muscle strength.

Grade	Description
0	No movement or detectable muscle contraction.
1	No movement, but a flicker or slight muscle contraction may be felt.
2	With arms extended in front of the body, the patient lifts her head and cervical spine off the surface, but the scapulae remain down.
3	With arms extended in front of the body, the patient lifts the lower angles of the scapulae from the surface.
4	With arms crossed over the chest, the patient lifts the lower angles of the scapulae from the surface.
5	With hands placed beside the ears, the patient lifts the lower angles of the scapulae from the surface.

**Table 2:** Illustration of the two exercise programs.

Component	Group A: Mobile Application (JustFit)	Group B: Traditional Exercise Program
Intervention Type	Individualized, technology-assisted program	Standardized, protocol-based program
Duration and Frequency	30 min/session, 3 times/week for 8 weeks	30 min/session, 3 times/week for 8 weeks
Supervision	Weekly supervised follow-up sessions	Weekly supervised follow-up sessions
Program Customization	Algorithm-driven customization based on user input	Therapist-based supervised protocol
Input Parameters	Motivation, main goal, area of focus, gender, age, height, current weight, goal weight, workout place, workout type, workout level, history of injury, activity level, fitness level	Not applicable
Participant Profile (in this study)	Goal: Get in shape, improve appearance, and focus on fitness and abdominal muscles (flat belly). Injury: None. Activity: Light. Active. Fitness: Beginner. Preference: Yoga mat, no equipment, slightly sweaty.	Not applicable
Exercise Components	Customized abdominal strengthening program including variations of crunches, abdominal braces, bridges, leg raises, flutter kicks, planks, side planks, bird-dog, dead bug, and standing core movements.	Standardized protocol consisting of head lifts with posterior pelvic tilts, isometric abdominal contractions (crook-lying), rotational curl-ups, abdominal curl-ups, pelvic bridge, side-bridge, and plank exercises.
Session Structure	8–12 exercises per session. Alternating time-based (15–45 s) and repetition-based (10–20 repetitions) tasks. Rest intervals: 10–20 s.	Fixed sequence of exercises: each exercise is held for 5 s, repetition-based (15 repetitions). Rest intervals for 10 s.
Progression Mechanism	Algorithm-driven: Gradual increase in training load by: extending exercise duration (e.g., 15 → 45 s), increasing repetitions (e.g., 10 → 20 repetitions), increasing sets (e.g., 4 → 10 sets), introducing more challenging positional variations.	Gradual increase in training load by: extending exercise duration (e.g., 5 → 15 s), increasing repetitions (e.g., 15 → 30 repetitions), increasing sets (e.g., 2 → 3 sets), introducing more challenging positional variations.
Delivery Method	Mobile application (JustFit, ENERJOY PTE. Ltd., Singapore) with reminders and alarms.	Traditional instruction and demonstration.

**Table 3:** Comparison of the physical features between the 2 groups.

Items	Physical Features of the Participants	
	BMI Measured in kg/m^2^ (Mean ± SD)	Age Measured in Years (Mean ± SD)
Group A (n = 20)	27.31 ± 1.58 95%CI: [26.57–28.05]	28.45 ± 1.79 95%CI: [26.85–27.97]
Group B (n = 20)	27.41 ± 1.19 95%CI: [27.61–29.29]	28.55 ± 2.78 95%CI: [27.25–29.85]
MD	−0.10	−0.10
Effect size	−0.072	−0.042
*p*-value	0.823	0.893
Significance	NS	NS

MD: Mean difference; *p*-value: probability value; NS: non-significant.

**Table 4:** Comparisons of the ultrasonographic findings of inter-recti separation.

Ultrasonographic Findings	Medians and IQR	Mean Ranks	*p*-Value	Medians and IQR	Mean Ranks	*p*-Value	*p*-Value Between Groups A&B
	Group A			Group B			
Pre-treatment IRD above the umbilicus	1.32 (0.09)	20.80		1.32 (0.12)	20.20		0.870
Post-treatment IRD above the umbilicus	0.89 (0.08)	10.78	<0.001 *	1.10 (0.10)	30.23	<0.001 *	<0.001 *
Pre-treatment IRD below the umbilicus	1.58 (0.30)	22.98		1.44 (0.15)	18.03		0.178
Post-treatment IRD below the umbilicus	1.19 (0.28)	17.65	<0.001 *	1.30 (0.13)	23.35	<0.001 *	0.122

IQR: interquartile range; *p*-value: probability value; *: significant.

**Table 5:** Comparisons of manual muscle testing (MMT) variables.

Manual Muscle Testing (MMT) for Abdominal Muscles	Medians and IQR	Mean Ranks	*p*-Value	Medians and IQR	Mean Ranks	*p*-Value	*p*-Value Between Both Groups A&B
	Group A			Group B			
Pre-treatment MMT for Rectus Abdominis	4 (0)	20.50		4 (0)	20.50		1.000
Post-treatment MMT for Rectus Abdominis	8 (0)	30.50	<0.001 *	7 (0)	10.50	<0.001 *	<0.001 *
Pre-treatment MMT for Abdominal Obliques	4 (0)	20.50		4 (0)	20.50		1.000
Post-treatment MMT for Abdominal Obliques	8 (0)	30.50	<0.001 *	7 (0)	10.50	<0.001 *	<0.001 *

IQR: interquartile range; *p*-value: probability value; *: significant.

**Table 6:** Comparisons of girth measurements at different levels.

Girth Measurements	Medians and IQR	Mean Ranks	*p*-Value	Medians and IQR	Mean Ranks	*p*-Value	*p*-Value Between the Two Groups A&B
	Group A			Group B			
Pre-treatment waist circumference	93.50 (10)	21.23		93.00 (8)	19.78		0.693
Post-treatment waist circumference	84.50 (9)	15.48	<0.001 *	88.00 (8)	25.53	<0.001 *	0.006 *
Pre-treatment umbilical circumference	104.50 (7)	22.25		103.00 (5)	18.75		0.339
Post-treatment umbilical circumference	95.00 (6)	15.80	<0.001 *	97.00 (3)	25.20	<0.001 *	0.010 *
Pre-treatment hip circumference	110.50 (11)	22.50		108.50 (5)	18.50		0.276
Post-treatment hip circumference	101.50 (13)	18.73	<0.001 *	103.50 (4)	22.28	<0.001 *	0.335

IQR: interquartile range; *p*-value: probability value; *: significant.

**Table 7:** Comparisons of the level of post-treatment satisfaction.

The Level of Satisfaction	Medians and IQR	Mean Ranks	Medians and IQR	Mean Ranks	*p*-Value Between Both Groups A&B
	Group A		Group B		
Post-treatment Satisfaction Level	1 (0)	25.00	0 (1)	16.00	0.003 *

IQR: interquartile range; *p*-value: probability value; *: significant.

**Table 8:** Correlation and regression of IRD post-treatment with other post-treatment measures.

Outcome	Spearman’s of IRD Above	*p*-Value (Above)	Spearman’s of IRD Below	*p*-Value (Below)	β IRD Above	β IRD Below	β Age	β BMI	R^2^
MMT rectus abdominis POST	−0.84	<<0.001 *	−0.25	0.12	−2.71	−0.04	−0.05	−0.12	0.73
MMT abdominal obliques POST	−0.84	<<0.001 *	−0.25	0.12	−2.71	−0.04	−0.05	−0.12	0.73
Waist circumference POST	0.43	0.006 *	0.08	0.63	10.89	1.58	0.45	1.88	0.15
Umbilical circumference POST	0.32	0.047 *	0.26	0.10	94.61	0.06	0.18	1.62	0.17
Hip circumference POST	0.19	0.25	−0.04	0.83	4.62	2.67	0.22	1.05	0.06
Level of Satisfaction	−0.30	0.06	−0.14	0.39	−0.88	−0.05	0.01	−0.01	0.09

Spearman’s ρ (rho): Spearman rank correlation coefficient, β: standardized regression coefficient, R^2^: coefficient of determination, *p*-value: probability value, *: Significant.

## Data Availability

The data that support the findings of this study and Supplementary Materials are available from the corresponding author upon reasonable request.

## Supplementary Materials

The following supporting information can be downloaded at: https://www.mdpi.com/article/10.3390/healthcare13233103/s1, File S1: Mobile App Abdominal Exercises; File S2. Ref. [26] was cited in File S2.

## Abbreviations

The following abbreviations are used in this manuscript:

DRA	Diastasis Rectus Abdominis
CS	Cesarean Section
IRD	Inter-Recti Distance
RCT	Randomized Controlled Trial
